# Dehydrogenation of Cycloalkanes over N-Doped Carbon-Supported Catalysts: The Effects of Active Component and Molecular Structure of the Substrate

**DOI:** 10.3390/nano11112846

**Published:** 2021-10-26

**Authors:** Jian Wang, He Liu, Shiguang Fan, Shuai Wang, Guanjun Xu, Aijun Guo, Zongxian Wang

**Affiliations:** State Key Laboratory of Heavy Oil Processing, College of Chemical Engineering, China University of Petroleum (East China), Qingdao 266580, China; j.wang@s.upc.edu.cn (J.W.); sgf@s.upc.edu.cn (S.F.); z20030074@s.upc.edu.cn (S.W.); z19030053@s.upc.edu.cn (G.X.); ajguo@upc.edu.cn (A.G.)

**Keywords:** hydrogen, Pt, cycloalkanes, dehydrogenation, molecular structure

## Abstract

Efficient dehydrogenation of cycloalkanes under mild conditions is the key to large-scale application of cycloalkanes as a hydrogen storage medium. In this paper, a series of active metals loaded on nitrogen-doped carbon (M/CN, M = Pt, Pd, Ir, Rh, Au, Ru, Ag, Ni, Cu) were prepared to learn the role of active metals in cycloalkane dehydrogenation with cyclohexane as the model reactant. Only Pt/CN, Pd/CN, Rh/CN and Ir/CN can catalyze the dehydrogenation of cyclohexane under the set conditions. Among them, Pt/CN exhibited the best catalytic activity with the TOF value of 269.32 h^−1^ at 180 °C, followed by Pd/CN, Rh/CN and Ir/CN successively. More importantly, the difference of catalytic activity between these active metals diminishes with the increase in temperature. This implies that there is a thermodynamic effect of cyclohexane dehydrogenation with the synthetic catalysts, which was evidenced by the study on the activation energy. In addition, the effects of molecular structure on cycloalkane dehydrogenation catalyzed by Pt/CN were studied. The results reveal that cycloalkane dehydrogenation activity and hydrogen production rate can be enhanced by optimizing the type, quantity and position of alkyl substituents on cyclohexane.

## 1. Introduction

Hydrogen is considered to be a promising alternative for widely used fossil fuels, because it has a high calorific value and is pollution-free [1,2]. However, hydrogen is flammable and explosive, which raises some safety concerns, thus hindering the large-scale commercial applications [3,4]. Liquid organic hydrogen carriers (LOHCs), storing hydrogen in their molecular structures, have been developed to overcome problems in safe storage and transportation of hydrogen [5,6,7,8,9,10]. As a kind of common LOHC, cycloalkanes hold high hydrogen storage density (5–8 wt%) and exhibit excellent chemical and thermal stability [11]. Furthermore, cycloalkanes are commonly used industrial chemicals which have realized long-distance transportation and large-scale storage in the chemical industry. However, the release of hydrogen in cycloalkanes is difficult because of the high thermodynamic limitation in C-H bond cleavage [5,12,13,14]. Extensive studies found that cycloalkane dehydrogenation with heterogeneous catalysts should be carried out in gas-phase at high temperature (above 300 °C) to obtain a high hydrogen production rate and cycloalkane conversion [11]. However, the high temperature in cycloalkane dehydrogenation might lead to catalyst deactivation and impure hydrogen [15,16]. Furthermore, the harsh reaction condition of cycloalkane dehydrogenation hinders the applications carried out under mild conditions, such as fuel cells [17].

Recently, “wet–dry multiphase conditions”, the dehydrogenation technology under reactive distillation conditions, has been developed to overcome the thermodynamic limitation in C-H bond cleavage, which can obtain high cycloalkane conversion and high hydrogen purity at low temperatures around 200 °C [18,19]. However, this technology is also accompanied by a low hydrogen production rate under mild conditions, which remains a key challenge in large-scale applications. One strategy is to develop a highly efficient catalyst for cycloalkane dehydrogenation under mild conditions. A series of Pt-based catalysts with excellent dehydrogenation performance under mild conditions (around 200 °C) have been intensively studied for cycloalkane dehydrogenation [18,19,20,21,22,23,24]. Li et al. investigated decalin dehydrogenation over 5 wt% Pt/p-CNFs catalyst under wet-dry multiphase conditions [19]. The result revealed that the Pt/p-CNFs catalyst exhibited the excellent dehydrogenation performance with the conversion of 46.32% and TOF of 732.4 mol H_2_/mol metal at 240 °C after 2 h. Hodoshima et al. studied the enhancement of dehydrogenation performance of Pt-based catalyst by the addition of Ir and W under wet–-dry multiphase conditions [23]. The decalin conversions over Pt/C, PtIr/C and PtW/C were 26.5%, 32% and 37.3% at 210 °C after 2.5 h, respectively. In our previous work, Pt/CN catalyst was employed in cyclohexane dehydrogenation with the cyclohexane conversion of 96.03% at 210 °C after 1.5 h [24]. In addition, many other metals (Pd, Ag, Ni, Cu, etc.) exhibited good catalytic performance in cycloalkanes dehydrogenation at elevated temperatures around 300 °C [16,25,26,27,28]. However, limited studies focused on the catalytic cycloalkane dehydrogenation under mild reaction conditions.

In addition to the catalysts, the molecular structures of cycloalkanes affect the dehydrogenation activity as well [5,22,29]. Kariya et al. revealed that dehydrogenation of methylcyclohexane over Pt/AC cloth at 298 °C exhibited better dehydrogenation activity comparable with that of cyclohexane at 330 °C [22]. Crabtree et al. studied the effect of amino groups on the dehydrogenation performance of cycloalkanes by theoretical calculation. The results showed that the temperature of hydrogen released by cycloalkanes gradually decreased with the increase in amino quantity [30]. Moreover, the enhancement of dehydrogenation activity by ethyl group in dodecahydro-N-ethylcarbazole dehydrogenation has been reported [31]. However, there is still a lack of systematic research on the effect of molecular structure on the dehydrogenation performance of cycloalkanes.

In this paper, we explored the role of metal (Pt, Pd, Rh and Ir) nanoparticles supported on nitrogen-doped carbon (CN) for cycloalkane dehydrogenation under wet–dry multiphase conditions with cyclohexane as the model reactant. Furthermore, the effects of type, quantity and position of alky substituents on cyclohexane for cycloalkanes dehydrogenation were investigated with Pt/CN as the model catalyst.

## 2. Experimental Section

### 2.1. Catalyst Preparation

#### 2.1.1. Synthesis of the Nitrogen-Doped Carbon Support (CN)

The nitrogen-doped carbon support (CN) was synthesized according to our previous report [24]. Briefly, dicyandiamide (1 g), glucose (1 g) and colloidal silica template (3 mL) were added in 40 mL of ultrapure water under stirring. Then, the mixture was heated at 120 °C until dried. The obtained yellow powder was then calcined under nitrogen atmosphere at 1000 °C for 3 h. After cooling to room temperature, the black solid was washed with 2 M of NaOH solution at 120 °C for 24 h. The precipitate was filtered via 220 nm organic diameter and washed with ultrapure water. CN support was collected after vacuum drying.

#### 2.1.2. Preparation of M/CN Catalysts

M/CN catalysts were prepared as follows [32]: CN support (400 mg) and a certain amount of metal salt (the metal quality was 5 wt% of CN) were added in 40 mL of ultrapure water successively. Subsequently, the above solution was sonicated for 30 min and then stirred at room temperature for 24 h. Finally, the mixture was reduced by 50 mg of sodium borohydride at 80 °C for 40 min. The products were washed with ultrapure water and collected after vacuum drying. The catalysts prepared from H_2_PtCl_6_·6H_2_O, Na_2_PdCl_4_, IrCl_3_·xH_2_O, Na_3_RhCl_6_, HAuCl_4_·4H_2_O, RuCl_3_, AgNO_3_, NiCl_2_·6H_2_O and Cu (NO_3_)_2_·3H_2_O are labeled as Pt/CN, Pd/CN, Ir/CN, Rh/CN, Au/CN, Ru/CN, Ag/CN, Ni/CN and Cu/CN, respectively.

### 2.2. Characterization of Catalysts

The X-ray diffraction (XRD) patterns and X-ray photoelectron spectroscopy (XPS) spectra were acquired on the X’ Pert Pro MPD X-ray diffractometer (Malvern Panalytical Ltd., Worcestershire, UK) and Thermo Fisher X-ray photoelectron spectrometer (Thermo Fisher Scientific, Waltham, MA, USA), respectively. The N_2_ adsorption/desorption isotherms of CN and the as-prepared catalysts were analyzed using a Micromeritics ASAP 2020 instrument (Micromeritics, Norcross, GA, USA) and calculated by BET method. The transmission electron microscope (TEM) images and high-resolution transmission electron microscope (HRTEM) images of the as-prepared catalysts were collected from a JEM-2100UHR transmission electron microscope (Jeol Ltd., Tokyo, Japan) at the accelerating voltage of 100 kV and 200 kV, respectively.

### 2.3. Catalytic Testing

The cycloalkanes dehydrogenation was performed in nitrogen atmosphere under the “wet–dry” multiphase conditions. Typically, 120 mg of M/CN catalyst and 340 mg of cycloalkanes were added to the reaction tube, which were placed in an oil bath to reach the required temperature. The volume of gas products was recorded online through the gas flowmeter. The compositions of gas and liquid products were determined by gas chromatography.

## 3. Results and Discussion

### 3.1. Effect of Metals

Various nitrogen-doped carbon supported metal catalysts (M/CN, M = Pt, Pd, Ir, Rh, Au, Ru, Ag, Ni, Cu) were prepared by fixing the mass ratio of the metal to the CN support as 5 wt%. The catalytic activity of M/CN catalyst in cycloalkane dehydrogenation have been tested with cyclohexane as the model compound. The cyclohexane dehydrogenation was performed at 180 °C (catalyst surface temperature) under the “wet–dry” multiphase conditions. Only Pt/CN, Pd/CN, Rh/CN and Ir/CN exhibited catalytic activity for cyclohexane dehydrogenation under the set conditions, and other synthetic catalysts were inactive (as shown in Table S1). Therefore, the four catalysts (Pt/CN, Pd/CN, Rh/CN and Ir/CN) for cyclohexane dehydrogenation were studied in detail. The active metal contents in Pt/CN, Pd/CN, Rh/CN and Ir/CN catalysts were 3.22 wt%, 2.64 wt%, 2.69 wt% and 2.32 wt%, respectively, which were determined by inductively coupled plasma atomic emission spectroscopy (ICP-AES) and listed in Table 1. In addition, the element analysis of C, N, O in the CN support are shown in Table S2.

**Figure 1:**
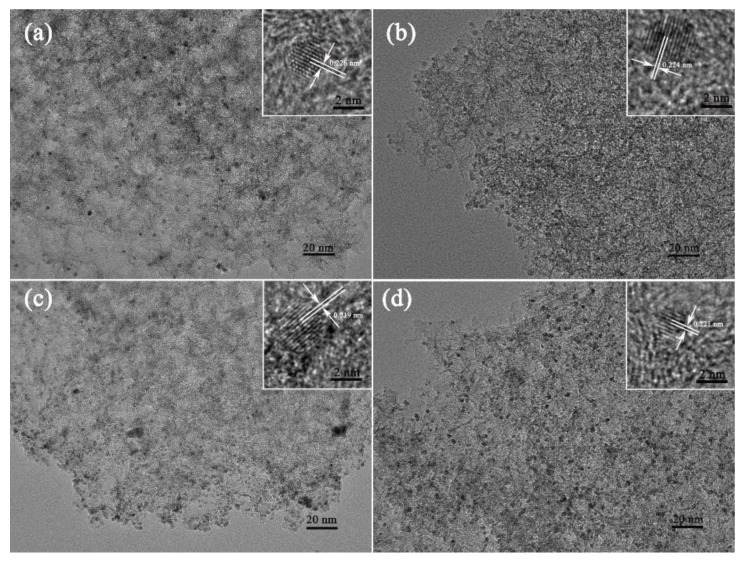
TEM and HRTEM (inset) images of (**a**) Pt/CN, (**b**) Pd/CN, (**c**) Rh/CN and (**d**) Ir/CN.

**Figure 2:**
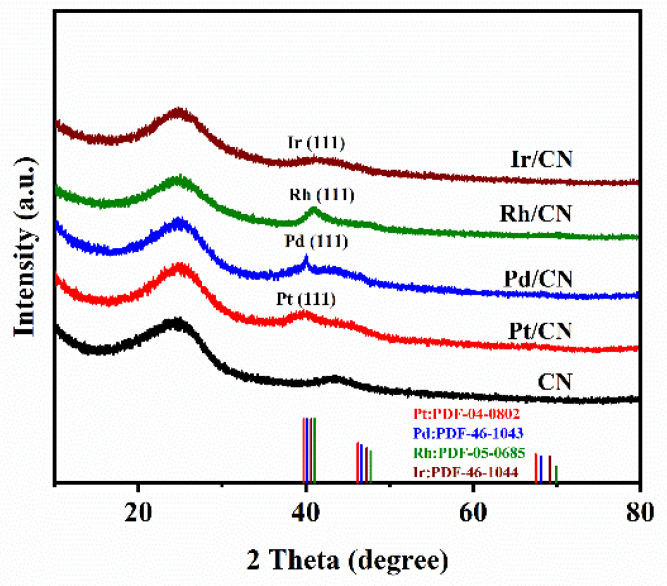
The XRD patterns of the CN support and M/CN (M = Pt, Pd, Rh and Ir) catalysts.

The morphologies of the Pt/CN, Pd/CN, Rh/CN and Ir/CN catalysts were investigated by TEM and HRTEM and presented in Figure 1. The TEM images of four catalysts display that ultrasmall metal nanoparticles disperse well on the CN support. Figure S1 depicts the size distributions of metal nanoparticles on Pt/CN, Pd/CN, Rh/CN and Ir/CN catalysts. The average particle sizes of Pt, Pd, Rh and Ir were calculated to be 2.07 ± 0.13, 2.46 ± 0.27, 2.5 ± 0.32 and 2.29 ± 0.34 nm, respectively. Obvious lattice fringes were observed in the HRTEM images. The interplanar distance of lattice fringes were calculated to be 0.226, 0.224, 0.219 and 0.221 nm, corresponding to (111) plane of Pt, Pd, Rh and Ir, respectively. XRD patterns of M/CN (M = Pt, Pd, Rh and Ir) are presented in Figure 2. Peaks of (111) plane of corresponding metal can be clearly observed in the XRD patterns of M/CN catalysts, which match well with the diffraction peaks of Pt (PDF-04-0802), Pd (PDF-46-1043), Rh (PDF-05-0685) and Ir (PDF-46-1044), respectively.

In addition, BET measurement was employed to explore surface areas and pore size distributions in M/CN (M = Pt, Pd, Rh and Ir) catalysts. As shown in Figure S2 and Table 1, the BET surface areas and pore sizes of the Pt/CN, Pd/CN, Rh/CN and Ir/CN catalysts are almost closed to each other. The values of BET surface area are distributed in 917.55–943.88 m^2^/g, with the pore size distribution of 10.53–10.70 nm. It implies that the similar BET surface areas and pore sizes in four catalysts will have similar reactive species transfer.

**Figure 3:**
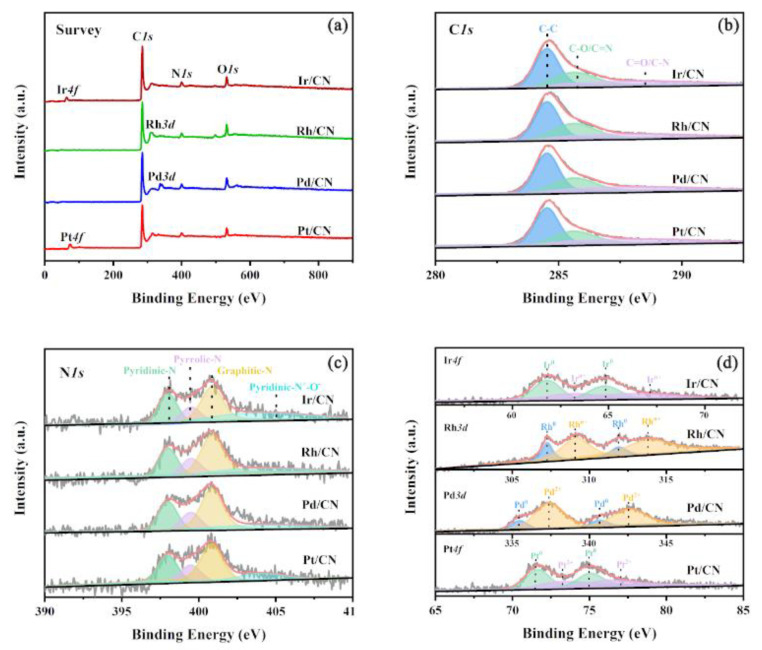
(**a**) XPS spectra, (**b**) C *1s* spectra, (**c**) N *1s* spectra and (**d**) Pt *4f*, Pd *3d*, Rh *3d* and Ir *4f* spectra of the M/CN (M = Pt, Pd, Rh and Ir) catalysts.

The active species compositions and chemical states of the synthetic samples have been determined by XPS. As shown in Figure 3a, signals of C *1s*, O *1s* and N *1s* are presented in the XPS spectra of Pt/CN, Pd/CN, Rh/CN and Ir/CN catalysts concurrently, which derive from the CN supports. The signals of Pt *4f*, Pd *3d*, Rh *3d* and Ir *4f* are also observed in their respective survey spectra. The high-resolution spectra of C *1s*, N *1s*, Pt *4f*, Pd *3d*, Rh *3d* and Ir *4f* were further studied and shown in Figure 3b–d. As shown in Figure 3b, the C species of four catalysts are almost the same, and the C 1s region is fitted into three peaks (C-C (284.60 eV), C-O/C=N (285.60 eV) and C=O/C-N (287.95 eV)) [33]. A similar situation occurs on the N *1s* spectra of M/CN (M = Pt, Pd, Rh and Ir) catalysts. As shown in Figure 3c, the four fitting peaks located at 398.0, 399.48, 400.84 and 403.39 eV belong to pyridinic N, pyrrolic N, graphitic N, and oxidized N, respectively [34]. The high-resolution spectra of active metals are present in Figure 3d. Pt *4f*_7/2_ and Pt *4f*_5/2_ peaks at 71.62 and 75.03 eV are attributed to the Pt^0^ species, whereas Pt *4f*_7/2_ and Pt *4f*_5/2_ peaks at 73.52 and 76.23 eV are attributed to the Pt^2+^ species [35]. The Pd *3d*_7/2_ and Pd *3d*_5/2_ of Pd/CN catalyst at 335.46 and 340.63 eV are attributed to the Pd^0^ species, while the peaks at 337.35 and 342.55 eV are attributed to the Pd^2+^ species [36,37]. As the fitting spectra of the Rh 3d region in Figure 3d, the copresence of metallic Rh state (Rh *3d*_7/2_ at 307.27 eV, Rh *3d*_5/2_ at 311.90 eV) and oxidized Rh state (Rh *3d*_7/2_ at 309.13 eV, Rh *3d*_5/2_ at 313.84 eV) on the surface of Rh/CN catalyst are exhibited [38]. Similarly, the fitting spectra of the Ir 4f region exhibit the copresence of metallic Ir state (Ir *4f*_7/2_ at 61.79 eV, Ir *4f*_5/2_ at 64.77 eV) and oxidized Ir state (Ir *4f*_7/2_ at 63.09 eV, Ir *4f*_5/2_ at 66.07 eV) on the surface of Ir/CN catalyst [39]. In summary, the differences in the electronic structure and chemical state of M/CN (M = Pt, Pd, Rh and Ir) catalysts mainly come from the active metals.

**Figure 4:**
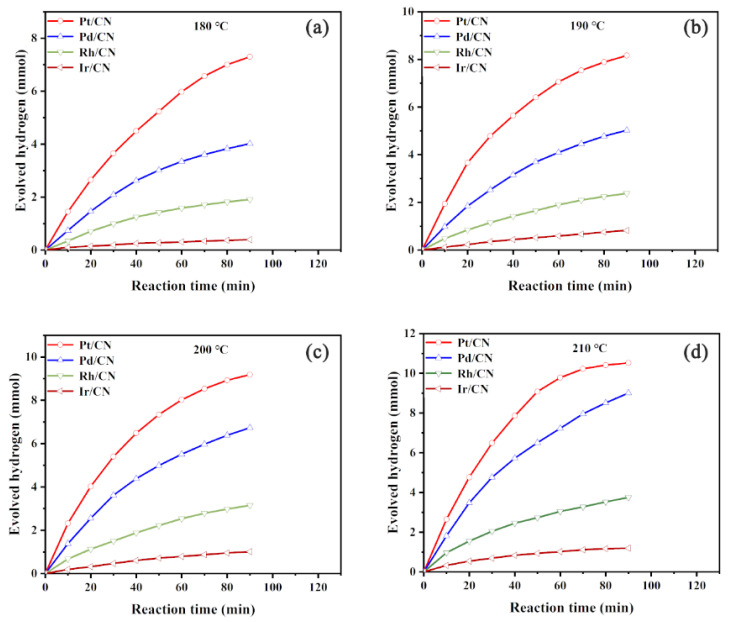
Hydrogen production curves of cyclohexane dehydrogenation with M/CN (M = Pt, Pd, Rh and Ir) catalysts at (**a**) 180 °C, (**b**) 190 °C, (**c**) 200 °C and (**d**) 210 °C.

**Figure 5:**
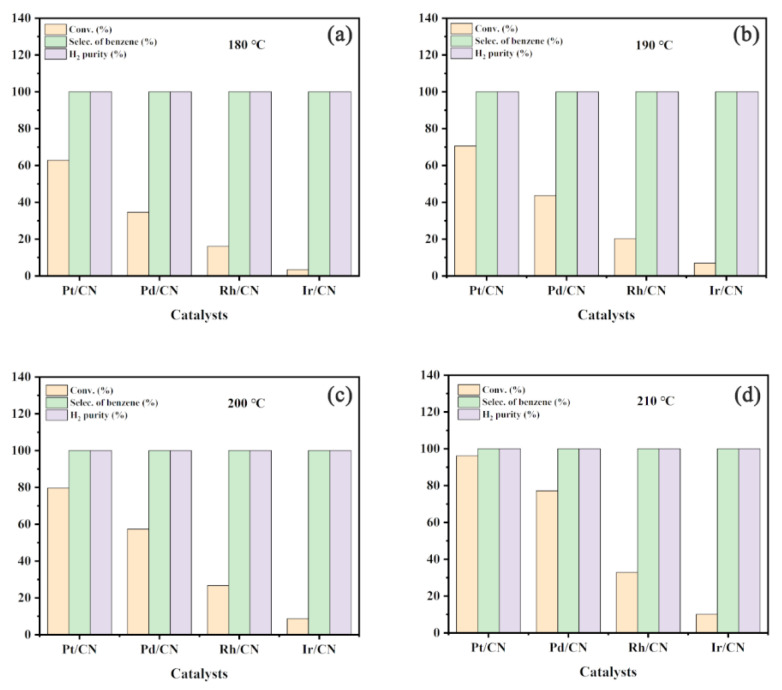
Cyclohexane conversions, selectivities of benzene and H_2_ purities with M/CN (M = Pt, Pd, Rh and Ir) catalysts at (**a**) 180 °C, (**b**) 190 °C, (**c**) 200 °C and (**d**) 210 °C.

The catalytic activities of M/CN (M = Pt, Pd, Rh and Ir) catalysts were evaluated for cyclohexane dehydrogenation at various reaction temperatures of 180–210 °C for 90 min. As shown in Figure 4, Pt/CN exhibits the best hydrogen production efficiency. The amount of hydrogen produced at 180 °C after 90 min with Pt/CN, Pd/CN, Rh/CN and Ir/CN catalysts are 7.30 mmol, 4.02 mmol, 1.91 mmol, 0.40 mmol, respectively. The hydrogen production efficiencies of all synthetic catalysts increased with the reaction temperature from 180 to 210 °C. As shown in Figure 5, the cyclohexane conversions of Pt/CN, Pd/CN, Rh/CN and Ir/CN catalysts at 180 °C are 62.83%, 34.58%, 16.09% and 3.37%, while the corresponding cyclohexane conversions at 210 °C are 96.22%, 77.16%, 32.85% and 10.15%, respectively. Moreover, the liquid and gas products of all synthetic catalysts were analyzed by GC. The results presented in Figure 5 indicate that only cyclohexane, benzene and H_2_ are detected, implying the highly selectivity of M/CN in cyclohexane dehydrogenation.

To further clarify the catalytic activities of active metals, the turnover frequencies (TOF) based on surface atoms of active metals of all synthetic catalysts for cyclohexane dehydrogenation were calculated according to the following two equations [40]:

(1) $$TOF\;\left(h^{-1},surface\right)=\frac{\frac{1}{3}\times rate\;of\;H_{2}\left(mol•h^{-1}\right)}{D\times moles\;of\;active\;metal\left(mol\right)}$$

where the rate of H_2_ is the rate at hydrogen production of 0.05 mmol, *D* is the dispersion of active metal. 

The dispersion of active metal (*D*) was calculated by equation:

(2) $$D\left(\%\right)=\frac{6\times C\times M\times 10^{9}}{\rho \times d\times N_{A}}\times 100$$

where *C* is the number of active metal surface atoms (Pt: 1.24 × 10^19^ atoms/m^2^, Pd: 1.27 × 10^19^ atoms/m^2^, Rh: 1.33 × 10^19^ atoms/m^2^, Ir: 1.30 × 10^19^ atoms/m^2^), *M* is the molar weight of active metal (Pt:195.08 g/mol, Pd: 106.42 g/mol, Rh: 102.90 g/mol, Ir: 192.20 g/mol), *ρ* is the density of active metal (Pt: 2.145 × 10^7^ g/m^3^, Pd:1.202 × 10^7^ g/m^3^, Rh: 1.241 × 10^7^ g/m^3^, Ir: 2.256 × 10^7^ g/m^3^), *d* is the average particle size of active metal, *N_A_* is the Avogadro constant (6.02 × 10^23^ mol^−1^).

**Figure 6:**
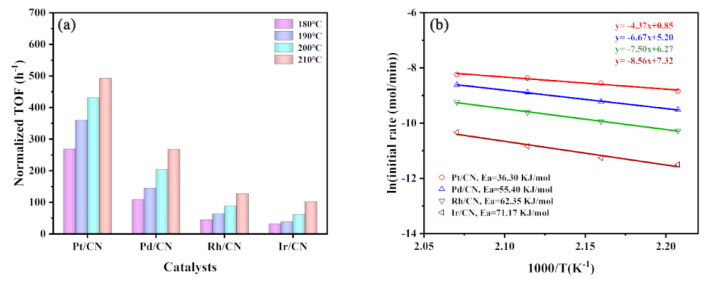
(**a**) TOF values at 180–210 °C and (**b**) Arrhenius plots of cyclohexane dehydrogenation.

As displayed in Figure 6a, Pt/CN exhibits the highest TOF values in the whole temperature range of 180–210 °C, followed by Pd/CN, Rh/CN and Ir/CN catalysts successively. Typically, the TOF value of Pt/CN, Pd/CN, Rh/CN and Ir/CN catalysts at the reaction temperature of 180 °C is 269.32, 108.78, 44.15, 31.73 h^−1^, respectively, with the corresponding TOF value at 210 °C of 492.52, 268.09, 127.39, 102.20 h^−1^, respectively. It indicates that the activity order of the M/CN catalysts on cyclohexane dehydrogenation is Pt/CN > Pd/CN > Rh/CN > Ir/CN. In addition, compared with other reported catalysts, Pt/CN and Pd/CN exhibit excellent hydrogen production performance (Table S3). Moreover, the difference of catalytic activity of catalysts is closely related to reaction temperature. The TOF value of Pt/CN catalyst was calculated to be 8.49 times that of Ir/CN catalyst at 180 °C, corresponding to be 4.82 times at 210 °C. It demonstrates that the difference of catalytic activity between different active metals diminishes with the increase in temperature.

To investigate the thermodynamic effects of cyclohexane dehydrogenation with M/CN catalysts, activation energies (Ea) were estimated by the Arrhenius equation [41,42]. As shown in Figure 6b, the Ea values were calculated and listed as follows: Pt/CN (36.30 kJ mol^−1^) < Pd/CN-2.3 (55.40 kJ mol^−1^) < Rh/CN (62.35 kJ mol^−1^) < Ir/CN (71.17 kJ mol^−1^). The results are consistent with the trend of TOF values of the M/CN catalysts on cyclohexane dehydrogenation.

### 3.2. Effect of Molecular Structure

**Figure 7:**
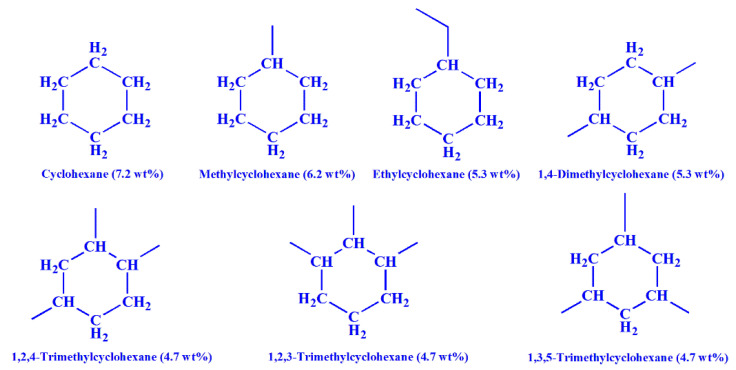
The molecular structure and hydrogen storage density of the chosen cycloalkanes.

In catalytic cycloalkane dehydrogenation, both active metals and the structure of the reactant will affect the catalytic performance. In this work, seven cycloalkanes with different alkyl substituent were selected to explore the influences of structure on cycloalkane dehydrogenation, which was carried out with Pt/CN as catalyst. The structure and hydrogen storage density of the chosen cycloalkanes are shown in Figure 7.

**Figure 8:**
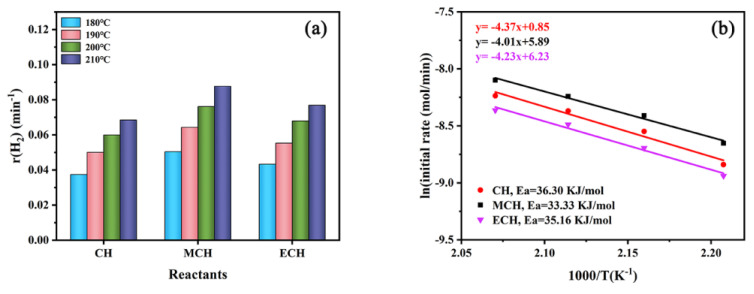
(**a**) r vales at 180–210 °C; (**b**) Arrhenius plots for CH, MCH and ECH dehydrogenation with Pt/CN catalyst.

Firstly, cyclohexane (CH), methylcyclohexane (MCH) and ethylcyclohexane (ECH) were employed to investigate the effect of the substituent group on cycloalkane dehydrogenation. As shown in Figure S3, the hydrogen production of CH dehydrogenation is the better than that of MCH and ECH at a whole temperature range of 180–210 °C after 90 min. However, the CH conversion is worse than that of MCH and ECH (Figure S4). The mismatch between hydrogen production and reactant conversion is due to the difference in hydrogen storage density of reactants. In order to eliminate the effect of hydrogen density of reactants, the relative rate of hydrogen production (*r*) was applied instead of TOF to compare the dehydrogenation performances of different cycloalkanes. The r value was calculated by the Equation (3).

(3) $$r\;\left(min^{-1}\right)=\frac{rate\;of\;H_{2}\left(mol•min^{-1}\right)}{moles\;of\;cycloalkanes\;\left(mol\right)}$$

where the rate of H_2_ is the rate at hydrogen production of 0.05 mmol. Figure 8a. The r value of MCH dehydrogenation is the biggest in the whole temperature range of 180–210 °C, followed by ECH and CH successively. It indicated that the dehydrogenation activities of the three cycloalkanes follow the order of MCH > ECH > CH. Significantly, the difference of dehydrogenation activity between different reactants decreases with the increase in temperature. Therefore, Ea of the three-cycloalkane dehydrogenation were estimated and shown in Figure 8b. The Ea values are listed as follows: MCH (33.33 kJ mol^−1^) < ECH (35.16 kJ mol^−1^) < CH (36.30 kJ mol^−1^), which are consistent with the trend of r values of the three-cycloalkane dehydrogenation. The results reveal that the addition of a methyl or ethyl group can improve the dehydrogenation activity of cycloalkanes, and the methyl group is more conducive to cycloalkane dehydrogenation than the ethyl group.

**Figure 9:**
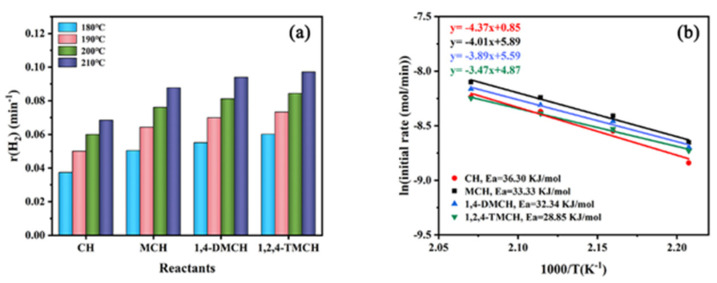
(**a**) r vales at 180–210 °C; (**b**) Arrhenius plots for CH, MCH, 1,4-TMCH and 1,2,4-TMCH dehydrogenation with Pt/CN catalyst.

Subsequently, the effect of quantity of methyl substituent on cycloalkane dehydrogenation was investigated with cyclohexane (CH), methylcyclohexane (MCH), 1,4-dimethylcyclohexane (1,4-DMCH) and 1,2,4-trimethylcyclohexane (1,2,4-TMCH) as model compounds. The dehydrogenation performances of selected cycloalkanes are displayed in Figures S5 and S6. A situation similar to that discussed above has emerged. The results of hydrogen production and reactant conversion are inconsistent. By comparing the r values as shown in Figure 9a, the dehydrogenation activity is in the following order: 1,2,4-TMCH > 1,4-DMCH > MCH > CH. Moreover, the same trend is observed in Ea of cycloalkane dehydrogenation shown in Figure 9b. The experimental results demonstrate that the increase in methyl substituent makes cycloalkanes release hydrogen more easily.

**Figure 10:**
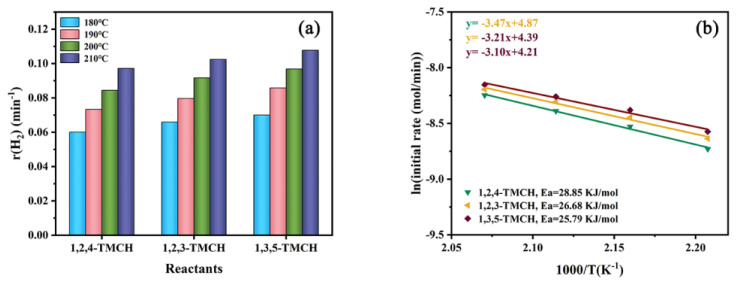
(**a**) r vales at 180–210 °C; (**b**) Arrhenius plots for 1,2,4-TMCH, 1,2,3-TMCH and 1,3,5-TMCH dehydrogenation with Pt/CN catalyst.

Finally, 1,2,3-trimethylcyclohexane (1,2,3-TMCH), 1,2,4-trimethylcyclohexane (1,2,4-TMCH) and 1,3,5-trimethylcyclohexane (1,3,5-TMCH) were selected to explore the effect of the substitution position. Because the hydrogen storage density of the three reactants is the same, the trend of hydrogen production efficiency (Figure S7) and reactant conversion (Figure S8) and r value (Figure 10a) of the three cycloalkanes are consistent. As shown in Figure 10a, the r values are listed as follows at the whole temperature range: 1,3,5-TMCH > 1,2,3-TMCH > 1,2,4-TMCH. With the increase in reaction temperature, the r values of the three cycloalkanes gradually approached. Furthermore, the Ea of the three-cycloalkane dehydrogenation exhibits the same trend (Figure 10b). The results demonstrate that the positions of methyl substituents affect the dehydrogenation performance.

## 4. Conclusions

In summary, we investigated the catalytic role of metal (Pt, Pd, Rh and Ir) nanoparticles supported on nitrogen-doped carbon using cyclohexane dehydrogenation as the model reaction. Pt/CN shows the best catalytic activity for cyclohexane dehydrogenation, and the dehydrogenation activity of the four metals is as follows: Pt > Pd > Rh > Ir. Moreover, seven cycloalkanes were selected to explore the role of molecular structure on cycloalkanes dehydrogenation. The results show that (1) adding alkyl substituents can improve the dehydrogenation activity of cycloalkanes, methyl is more conducive to cycloalkane dehydrogenation than ethyl; (2) the increased quantity of methyl substituent makes cycloalkanes release hydrogen more easily; (3) the dehydrogenation activity of cyclohexane is affected by the position of substituents. This study is helpful to understand the role of active metal and molecular structure on catalytic dehydrogenation of cycloalkanes and provides strategies for highly efficient catalytic cycloalkane dehydrogenation.

## Figures and Tables

**Table 1:** The physicochemical properties of the CN support and synthetic catalysts.

Sample	Metal (wt%) ^1^	Mean Size (nm) ^2^	Dispersion (%) ^3^	S_BET_ (m^2^/g) ^4^	V_total_ (cm^3^/g) ^4^	d_BJH_ (nm) ^4^
CN	-	-	-	965.17	2.53	10.70
Pt/CN	3.22	2.07 ± 0.13	54.30	917.55	2.35	10.55
Pd/CN	2.64	2.46 ± 0.27	45.55	929.55	2.37	10.57
Rh/CN	2.32	2.29 ± 0.34	48.21	943.88	2.40	10.53
Ir/CN	2.69	2.50 ± 0.32	43.97	935.79	2.40	10.58

^1^ analyzed by ICP-AES. ^2^ counted from TEM images. ^3^ calculated from the Equation (2). ^4^ collected from N_2_ adsorption/desorption isotherm.

## Data Availability

The data presented in this study are available on request from the corresponding author.

## Supplementary Materials

The following are available online at https://www.mdpi.com/article/10.3390/nano11112846/s1, Table S1: Dehydrogenation performance of cyclohexane over the synthetic catalysts at 180 °C; Table S2. The element analysis data of CN; Figure S1: Size distributions of metal nanoparticles on (a) Pt/CN, (b) Pd/CN, (c) Rh/CN and (d) Ir/CN; Figure S2: (a) N_2_ adsorption–desorption isotherms and (b) pore size distribution curves of the CN support and M/CN (M = Pt, Pd, Rh and Ir) catalysts; Table S3. Comparison of dehydrogenation performance of cycloalkanes reported in literature; Figure S3: hydrogen production curves of CH, MCH and ECH dehydrogenation at (a) 180 °C, (b) 190 °C, (c) 200 °C and 210 °C; Figure S4: cycloalkane conversions, selectivities and H_2_ purities of CH, MCH and ECH dehydrogenation at (a) 180 °C, (b) 190 °C, (c) 200 °C and (d) 210 °C; Figure S5: hydrogen production curves of CH, MCH, 1,4-DMCH and 1,2,4-TMCH at the temperatures of (a) 180 °C, (b) 190 °C, (c) 200 °C and 210 °C; Figure S6. Cycloalkane conversions, selectivities and H_2_ purities of CH, MCH, 1,4-DMCH and 1,2,4-TMCH dehydrogenation at (a) 180 °C, (b) 190 °C, (c) 200 °C and (d) 210 °C; Figure S7: hydrogen production curves of 1,2,4-TMCH, 1,2,3-TMCH and 1,3,5-TMCH dehydrogenation at the temperatures of (a) 180 °C, (b) 190 °C, (c) 200 °C and 210 °C; Figure S8: cycloalkane conversions, selectivities and H_2_ purities of 1,2,4-TMCH, 1,2,3-TMCH and 1,3,5-TMCH dehydrogenation at (a) 180 °C, (b) 190 °C, (c) 200 °C and (d) 210 °C.
