# Supplementary material for: Dehydrogenation of Cycloalkanes over N-Doped Carbon-Supported Catalysts: The Effects of Active Component and Molecular Structure of the Substrate
Source: Nanomaterials (Basel). 2021 Oct 26;11(11):2846. doi: 10.3390/nano11112846 (PMC8617684; doi:10.3390/nano11112846)
Supplement: Supplementary file 1 [file nanomaterials-11-02846-s001.zip › nanomaterials-1440851-supplementary.pdf]

Article

Dehydrogenation of Cycloalkanes over N-Doped Carbon-Supported Catalysts: The Effects of Active Component and Molecular Structure of the Substrate

Jian Wang †, He Liu *,†, Shiguang Fan, Shuai Wang, Guanjun Xu, Aijun Guo and Zongxian Wang *

State Key Laboratory of Heavy Oil Processing, College of Chemical Engineering, China University of Petroleum (East China), Qingdao 266580, China; j.wang@s.upc.edu.cn (J.W.); sgf@s.upc.edu.cn (S.F.); z20030074@s.upc.edu.cn (S.W.); z19030053 @s.upc.edu.cn (G.X.); ajguo@upc.edu.cn (A.G.)

* Correspondence: liuhe@upc.edu.cn (H.L.); heavyoil@upc.edu.cn (Z.W.); Tel.: +86-0532-86980607 (Z.W.); Fax: +86-0532-86981787 (Z.W.)

† These authors contributed equally to this work

Table S1. performance of cyclohexane over the synthetic catalysts at 180 °C

Sample	Hydrogen production (mmol)	cyclohexane conversion (%)
Pt/CN	7.30	62.83
Pd/CN	4.02	34.58
Rh/CN	1.91	16.09
Ir/CN	0.40	3.37
Au/CN	0	0
Ag/CN	0	0
Ni/CN	0	0
Cu/CN	0	0

Table S2. The element analysis data of CN

Element	Content (wt %)
**C**	73.27
O	7.86
N	18.87

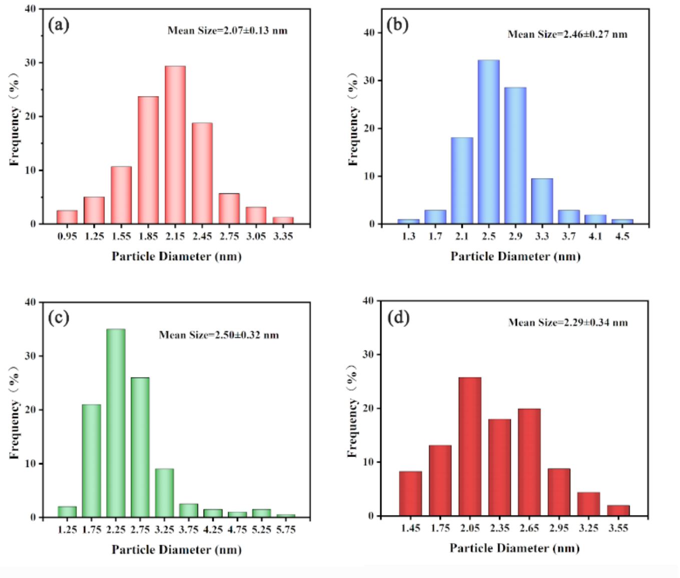

Figure S1. Size distributions of metal nanoparticles on (**a**) Pt/CN, (**b**) Pd/CN, (**c**) Rh/CN and (**d**) Ir/CN.

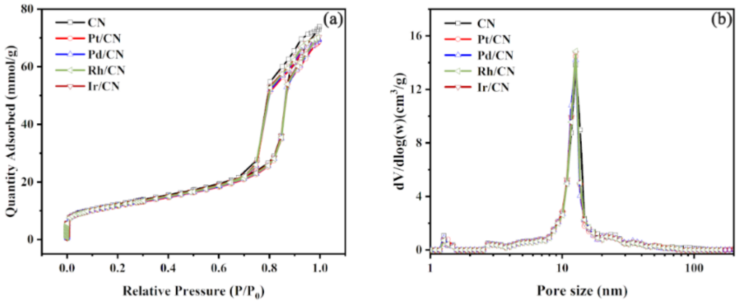

Figure S2. (**a**) N_2 adsorption–desorption isotherms and (**b**) pore size distribution curves of the CN support and M/CN (M = Pt, Pd, Rh and Ir) catalysts.

Tale S3. Comparison of dehydrogenation performance of cycloalkanes reported in literature

Reactant	Catalysts	Temp. (℃)	H_2 evolution rate (mmol/g_{metal}/min)	Ref.
Cyclohexane	10 wt.% Pt/CFF-1500S	260	98	[1]
Cyclohexane	2 wt% Pt/Alumina	315	29	[2]
Decalin	3 wt% Pt/C	210	45.4	[3]
Tetralin	5 wt% Pt/AC	210	103	[4]
Decalin	5 wt% Pt/AC	210	12.35	[5]
Decalin	Pt-W/AC (5 wt% metal)	210	42.31	[5]
Decalin	Pt–Ir/AC (5 wt% metal)	210	25.2	[5]
Decalin	1 wt% Pt/XC-72	240	18.45	[6]
Decalin	5 wt% Pt/p-CNFs	240	37.54	[6]
Decalin	5 wt% Pd/p-CNFs	240	8.98	[6]
Cyclohexane	3.22 wt% Pt/CN	180	37.48	This work
Cyclohexane	3.22 wt% Pt/CN	210	68.54	This work
Cyclohexane	2.64 wt% Pd/CN	180	23.28	This work
Cyclohexane	2.64 wt% Pd/CN	210	57.37	This work
1.3.5-Trimethylcyclohexane	3.22 wt% Pt/CN	180	48.90	This work
1.3.5-Trimethylcyclohexane	3.22 wt% Pt/CN	210	74.44	This work

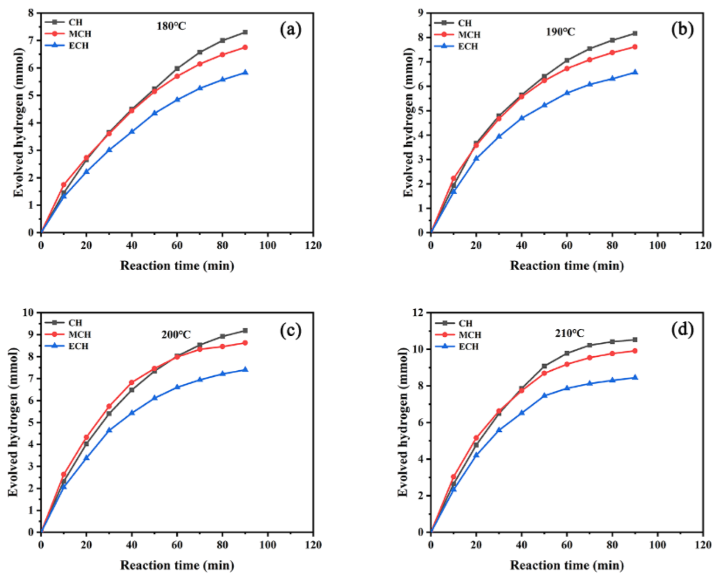

Figure S3. Hydrogen production curves of CH, MCH and ECH dehydrogenation at (**a**) 180 °C, (**b**) 190 °C, (**c**) 200 °C and (**d**) 210 °C.

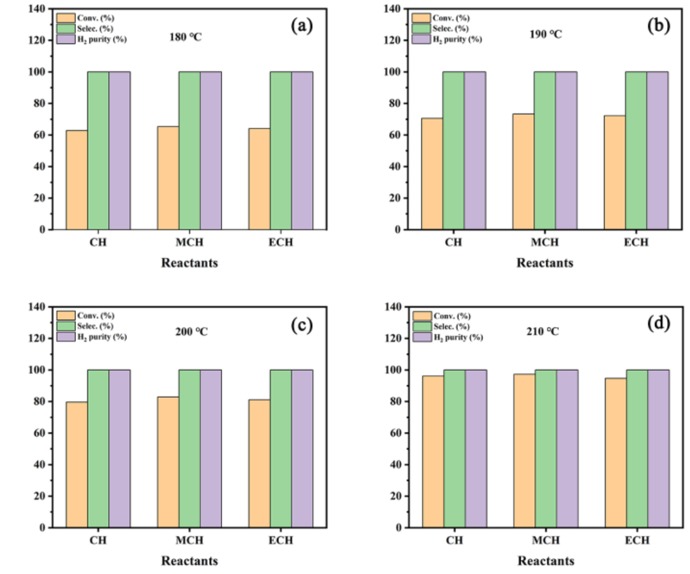

Figure S4. Cycloalkane conversions, selectivities and H_2 purities of CH, MCH and ECH dehydrogenation at (**a**) 180 °C, (**b**) 190 °C, (**c**) 200 °C and (**d**) 210 °C.

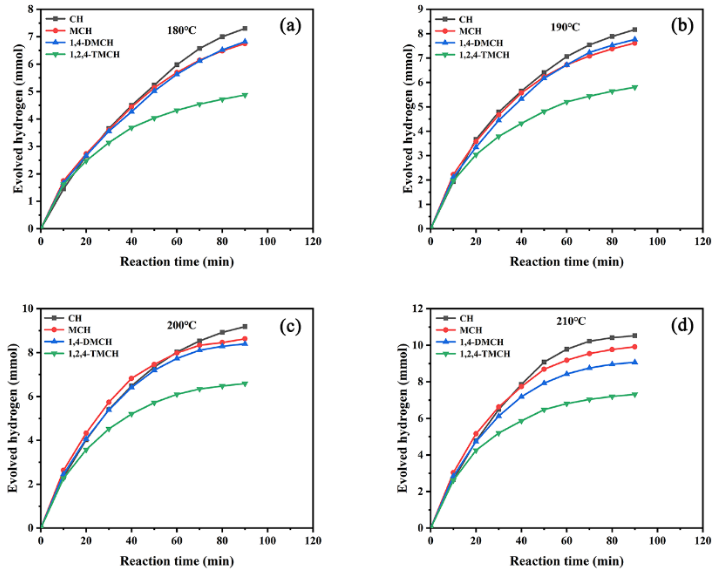

Figure S5. Hydrogen production curves of CH, MCH, 1,4-DMCH and 1,2,4-TMCH at the temperatures of (**a**) 180 °C, (**b**) 190 °C, (**c**) 200 °C and (**d**) 210 °C.

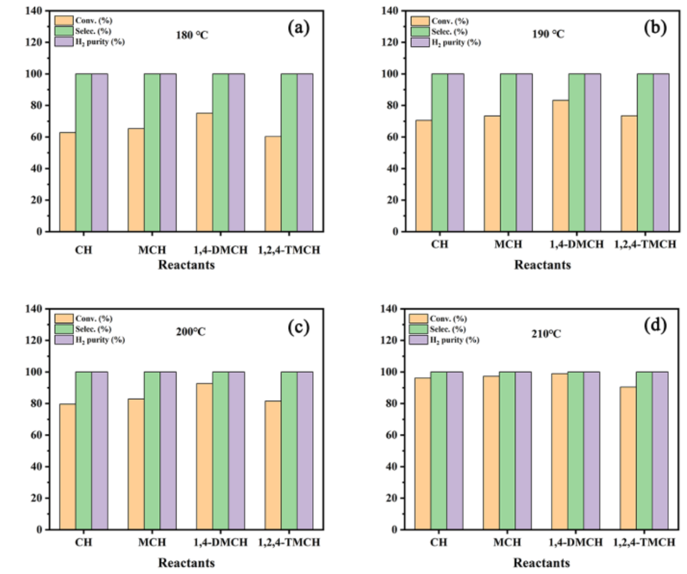

Figure S6. Cycloalkane conversions, selectivities and H_2 purities of CH, MCH, 1,4-DMCH and 1,2,4-TMCH dehydrogenation at (**a**) 180 °C, (**b**) 190 °C, (**c**) 200 °C and (**d**) 210 °C.

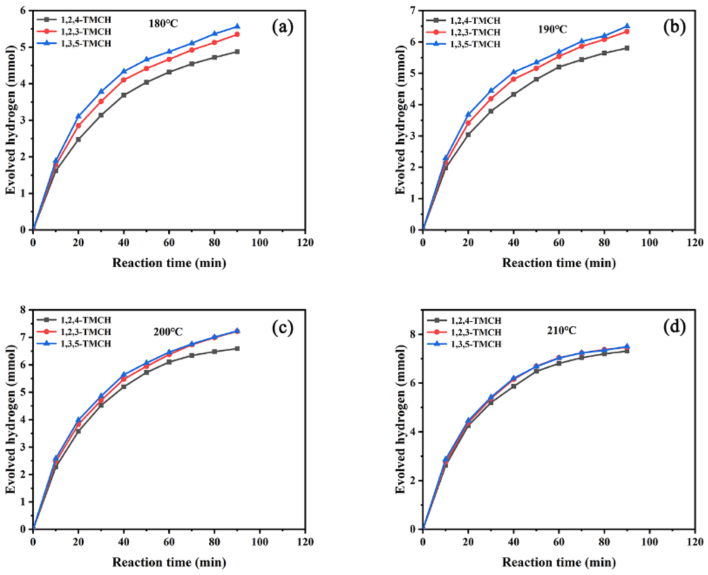

Figure S7. Hydrogen production curves of 1,2,4-TMCH, 1,2,3-TMCH and 1,3,5-TMCH dehydrogenation at the temperatures of (**a**) 180 °C, (**b**) 190 °C, (**c**) 200 °C and (**d**) 210 °C.

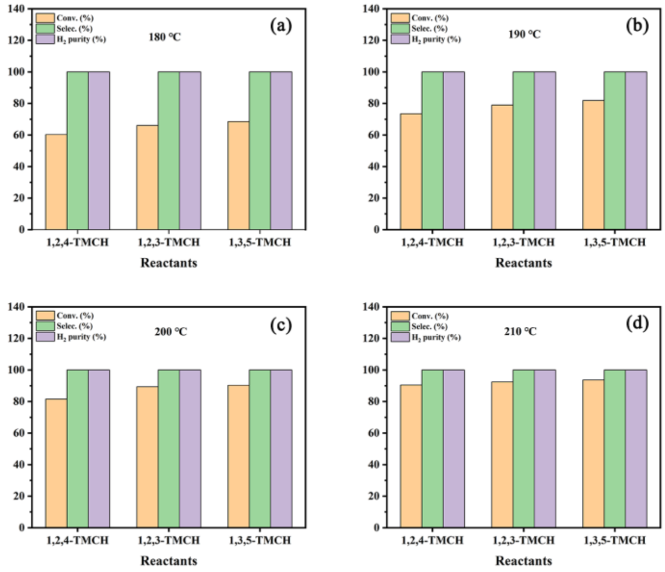

Figure S8. Cycloalkane conversions, selectivities and H_2 purities of 1,2,4-TMCH, 1,2,3-TMCH and 1,3,5-TMCH dehydrogenation at (**a**) 180 °C, (**b**) 190 °C, (**c**) 200 °C and (**d**) 210 °C.

Reference

1. Kariya, N.; Fukuoka, A.; Utagawa, T.; Sakuramoto, M.; Goto, Y.; Ichikawa, M., Efficient hydrogen production using cyclohex-ane and decalin by pulse-spray mode reactor with Pt catalysts. *Appl. Catal. A-Gen*. **2003**, 247, 247–259.
2. Kariya, N.; Fukuoka, A.; Ichikawa, M., Efficient evolution of hydrogen from liquid cycloalkanes over Pt-containing catalysts supported on active carbons under "wet–dry multiphase conditions". *Appl. Catal. A-Gen.* **2002,** 233, 91–102.
3. Gihoon, L.; Yeojin, J.; Bong-Geun, K.; Jin, S.; Heondo, J.; Hyon, B.; Ji, C., Hydrogen production by catalytic decalin dehydrogenation over carbon-supported platinum catalyst: Effect of catalyst preparation method. Catalysis Communications **2015**, 67, 40–44.
4. Hodoshima, S.; Hiroaki, N.; Yasukazu, S., Efficient hydrogen supply from tetralin with superheated liquid-film-type catalysis for operating fuel cells. *Appl. Catal. A-Gen.* **2005**, 292, 90–96.
5. Hodoshima, S.; Arai, H.; Saito, Y., Liquid-film-type catalytic decalin dehydrogeno-aromatization for long-term storage and long-distance transportation of hydrogen. *Int. J. Hydrogen Energ.* **2003**, 28, 197–204.
6. Li, P.; Huang, Y.; Chen, D.; Zhu, J.; Zhao, T.; Zhou, X., CNFs-supported Pt catalyst for hydrogen evolution from de-calin. *Catal. Commun*. **2009**, 10, 815–818.
